# Adherence to guideline recommendations for management of clinical T1 renal cancers in the Netherlands: a population-based study

**DOI:** 10.1007/s00345-016-1841-3

**Published:** 2016-05-13

**Authors:** Katja K. H. Aben, Susanne Osanto, Christina A. Hulsbergen-van de Kaa, Patricia M. Soetekouw, Daphne Stemkens, Axel Bex

**Affiliations:** Netherlands Comprehensive Cancer Organisation, P.O. Box 19079, 3501 DB Utrecht, The Netherlands; Radboud Institute for Health Sciences, Radboud University Medical Centre, P.O. Box 9101, 6500 HB Nijmegen, The Netherlands; Department of Oncology, Leiden University Medical Centre, P.O. Box 9600, 2300 RC Leiden, The Netherlands; Department of Pathology, Radboud University Medical Centre, P.O. Box 9101, 6500 HB Nijmegen, The Netherlands; Department of Oncology, Maastricht University Medical Centre, P.O. Box 5800, 6202 AZ Maastricht, The Netherlands; Department of Urology, Netherlands Cancer Institute, P.O. Box 90203, 1006 BE Amsterdam, The Netherlands; Netherlands Comprehensive Cancer Organisation, P.O. Box 1281, 6501 BG Nijmegen, The Netherlands

**Keywords:** Population-based, Cancer registry, Guideline adherence, Kidney cancer, Partial nephrectomy

## Abstract

**Purpose:**

For decades, small renal cancers are treated by radical nephrectomy (RN). Current guidelines recommend partial nephrectomy (PN) to preserve renal function and minimize cardiovascular comorbidity. As adherence to guidelines is largely unknown and international comparison to evaluate quality of health care is lacking, an pre-specified guideline evaluation of quality indicators concerning management of cT1 renal cancers was performed.

**Methods:**

We performed a cohort study including patients with cT1 renal cancer between 2010 and 2014, identified through the Netherlands Cancer Registry. Time trends and variation in treatment were described. Factors associated with PN in cT1a and laparoscopic RN in cT1b were evaluated with logistic regression analyses.

**Results:**

An increase in nephron-sparing treatment strategies (NSS) of cT1a patients (*N*_total_ = 2436) was observed; in 2014, 67 % underwent NSS (62 % PN and 5 % thermal ablation). Age, a non-central tumor localization and being treated in a high-volume hospital were associated with PN. Although NSS were applied more frequently over time, the majority (70 %) of cT1b patients (*N*_total_ = 2205) underwent RN in 2014, mainly performed laparoscopically. Increasing tumor size, tumor localization in the right kidney and being treated in a university hospital were associated with a lower probability of a laparoscopic RN versus open. Treatment in a high-volume hospital was associated with a higher probability of laparoscopic RN.

**Conclusions:**

Dutch patients with cT1 renal cancer are predominantly treated according to current guidelines. Data of this pre-specified quality indicator analysis of a urological national guideline may serve as a model for international comparison of treatment of cT1 renal cancers.

**Electronic supplementary material:**

The online version of this article (doi:10.1007/s00345-016-1841-3) contains supplementary material, which is available to authorized users.

## Introduction

Partial nephrectomy (PN) is recommended as treatment of choice for small renal cancers. The paradigm shift from radical nephrectomy (RN) to PN is based on a prospective randomized trial [1] which demonstrated similar oncological outcomes for both. In addition, several retrospective observational studies revealed comparable outcomes [2–5]. PN preserves renal function [2, 3, 6] and by this minimizes cardiovascular comorbidity [7, 8] and chronic kidney disease [2, 4].

The guidelines of the American Urological Association (AUA) from 2009 [9] and European Association of Urology (EAU) from 2014 [10] (http://www.uroweb.org) recommend PN for small kidney tumors (cT1a/cT1b, ≤7 cm) whenever possible. The Dutch guideline from 2010 (www.oncoline.nl) recommends PN for cT1a (≤4 cm) and states that for cT1b (>4 to ≤7 cm) RN is no longer the standard. All guidelines further mention that other nephron-sparing strategies (NSS) like thermal ablation (i.e., cryosurgery or radiofrequency ablation) are alternatives if PN is no option, due to technical reasons (e.g., anatomically complex masses) or high risk of surgical complications (e.g., in case of severe comorbid conditions). Finally, active surveillance may be considered, particularly in patients with cT1 tumors with a short life expectancy or severe comorbidities. Prospective cohort studies have shown acceptable outcomes in this patient group although the growth rate remains unpredictable [11].

Population-based studies from the USA including patient data until 2008 observed that, despite an increase in PN, RN remained the preferred procedure in almost 70 % of all kidney cancer patients [12, 13]. It was argued that the introduction of laparoscopic RN may have been favored as minimally invasive approach over the more complex PN which, before the advent of robot-assisted surgery, was frequently performed as open procedure. Generally, adherence to renal cancer guidelines is unknown which afflicts international comparison of healthcare quality.

Aim of this study was to evaluate adherence to recommendations in the Dutch guideline and to evaluate current management and variation in clinical practice. Based on a set of pre-specified quality indicators which included management of small renal cancers, we performed a population-based study using the Netherlands Cancer Registry (NCR).

## Patients and methods

The 2010 update of the Dutch Renal Cell Carcinoma guideline included an agreement to test its adherence based on pre-specified quality parameters. One of the parameters was management of cT1 renal cancers. Patients diagnosed with a clinical T1 renal cancer (International Classification of Diseases-Oncology (ICD-O), 3rd Edition code: C64) were selected from the NCR held by the Netherlands Comprehensive Cancer Organization (IKNL). Since 1989, all cancer diagnoses are recorded in the NCR and its completeness is estimated at approximately 95 % [14]. Patients diagnosed between 2010 and 2014 and age at diagnosis of ≥18 years were included. Patients without pathological confirmation of a renal cancer subtype [15] were excluded. Patient, tumor and treatment characteristic were retrieved from the NCR. All data available in the NCR are collected by review of medical files by well-trained registration staff in a uniform and standardized way. In case data were incomplete, an additional effort was made to complete the data by consulting the primary medical files again.

Treatment was categorized into four groups: PN, RN, thermal ablation and no treatment/active surveillance/other treatments. No distinction could be made for active surveillance versus no treatment; both were classified as ‘no treatment.’ Hospitals were grouped according to type: university hospitals, ‘top clinical’ hospitals and community hospitals). University hospitals, including the Netherlands Cancer Institute, represent nine large to midsize hospitals which offer specialized care and are responsible for the training of medical specialists. The ‘top clinical’ hospitals include large- to midsize hospitals which provide, next to standard care, specialized care in specific areas, are not affiliated with a university but involved in the training of medical specialists. The community hospitals include all other, mostly smaller-size, hospitals and provide standard care. Patients treated in foreign hospitals were excluded from all hospital specific analyses. Hospitals were also grouped according to oncological nephrectomy volume for renal cancer (<10, 10–19 and ≥20 procedures/year) as the national guideline recommends a minimum of 10 nephrectomy procedures.

With descriptive analyses, we provide insight into treatment trends over time between 2010 and 2014 for cT1a and cT1b renal cancer, stratified by age (<70 vs. ≥70 years at diagnosis. Five-year relative survival rates and 95 % confidence intervals (95 % CIs) are calculated for different treatment modalities. Follow-up was completed until January 1, 2015. We also compared differences in treatment between different types of hospitals for recently diagnosed patients (2012–2014). Uni- and multivariable logistic regression analyses were performed to identify factors associated with PN versus RN for patients diagnosed with cT1a cancer and factors associated with laparoscopic versus open RN for patients diagnosed with cT1b cancer in 2012–2014.

Comparative analyses of hospital stay (time in days between nephrectomy and discharge) and postoperative mortality (mortality within 30 days after surgery), after PN versus RN by tumor stage, were performed using a Wilcoxon-rank test and Chi-square test, respectively.

All analyses were performed with SAS 9, SAS institute Inc 2011, Cary, NC, USA. The study was approved by the internal review board of IKNL.

## Results

**Fig. 1:**
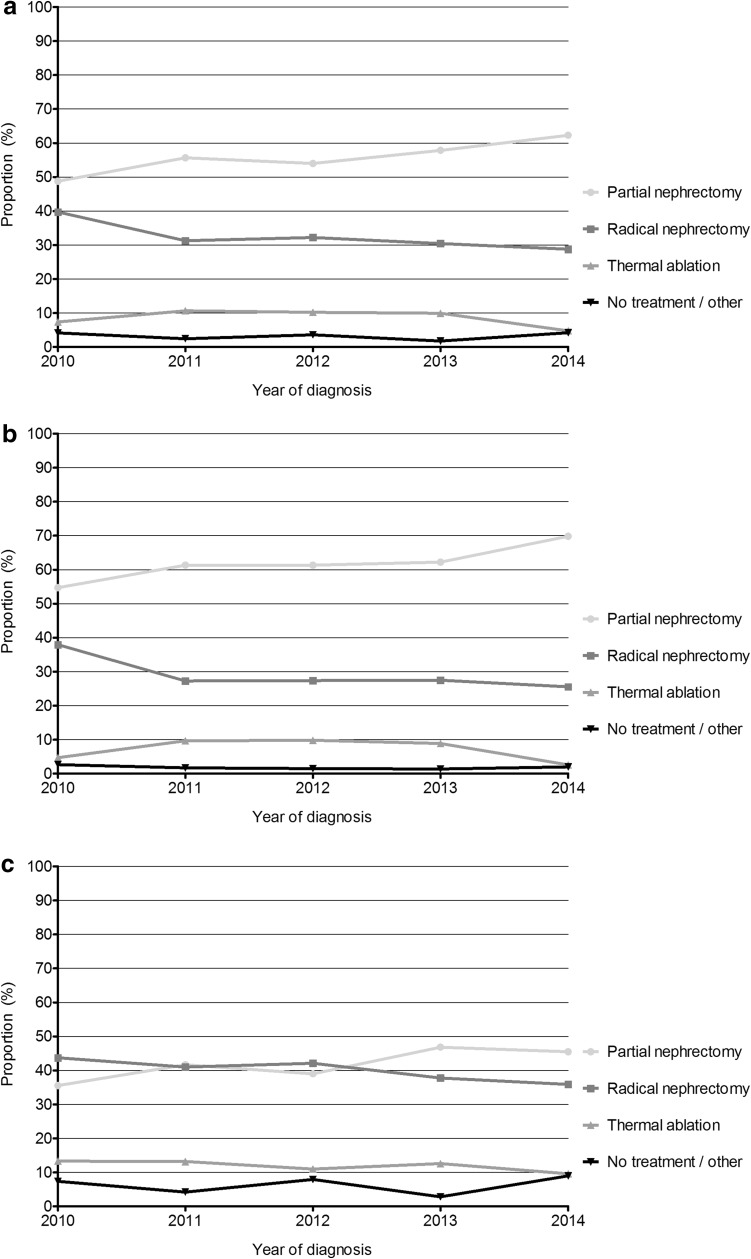
**a** Distribution of treatment modalities in cT1a renal cancer patients over time (2010–2014). **b** distribution of treatment modalities in cT1a renal cancer patients younger than 70 years over time (2010–2014). **c** distribution of treatment modalities in cT1a renal cancer patients of 70 years or older over time (2010–2014)

Overall 2436 patients with a cT1a renal cancer were diagnosed between 2010 and 2014. Subtypes were 68 % clear cell, 15 % papillary, 6 % chromophobe, 0.3 % sarcomatoid, 9 % unspecified and 1 % other. Figure 1a–c shows the proportion of cT1a patients by different treatment modalities. Over time, a clear increase in PN was observed. In 2014, 62 % underwent PN, 29 % RN, 5 % had thermal ablation, and only 4 % were not treated or followed an active surveillance protocol. Large differences were observed between patients ≥70 and <70 years. Thermal ablation as alternative NSS is predominantly used in older patients, and no clear increase since 2010 was observed. The overall 5-year relative survival rate of patients with cT1a was 93 % (95 % CI 89.6–95.8 %). Patients with PN or thermal ablation had a similar 5-year survival of 95.3 % (95 % CI 90.7–98.7 %) and 95.3 % (95 % CI 84.1–102.3 %), respectively. Patients with RN had a slightly worse survival of 92.9 % (95 % CI 87.1–97.3 %). Due to small numbers, the 5-year survival of patients with no active treatment could not be calculated.

**Fig. 2:**
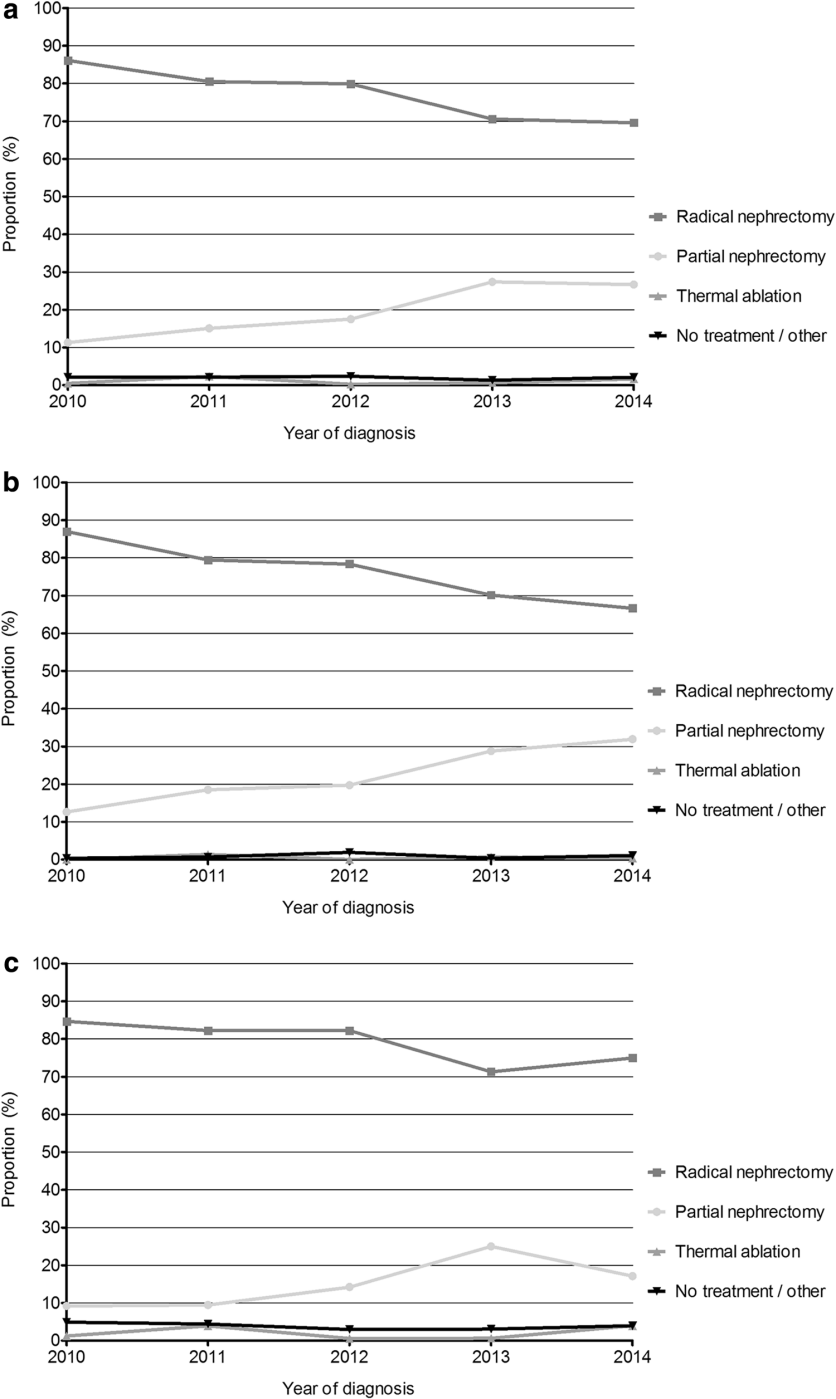
**a** Distribution of treatment modalities in cT1b renal cancer patients over time (2010–2014). **b** Distribution of treatment modalities in cT1b renal cancer patients younger than 70 years over time (2010–2014). **c** Distribution of treatment modalities in cT1b renal cancer patients of 70 years or older over time (2010–2014)

In total, 2205 patients with a cT1b renal cancer were diagnosed. Histological subtypes were 73 % clear cell, 11 % papillary, 5 % chromophobe, 0.5 % sarcomatoid, 8.5 % unspecified and 1 % other. NSS were applied more frequently over time for cT1b renal cancer (Fig. 2a–c), but RN remained the preferred treatment. In 2014, 70 % of all T1b patients underwent a RN and this proportion was largely independent of age. The overall 5-year relative survival rate of patients with cT1b was 87.3 % (95 % CI 83.3–90.8 %). Patients with PN had a 5-year survival of 93.1 % (95 % CI 80.4–100.5 %). Patients with RN had a slightly worse survival of 88.8 % (95 % CI 84.6–92.3 %). Due to small numbers, the 5-year survival of patients with thermal ablation or no active treatment could not be calculated.

**Fig. 3:**
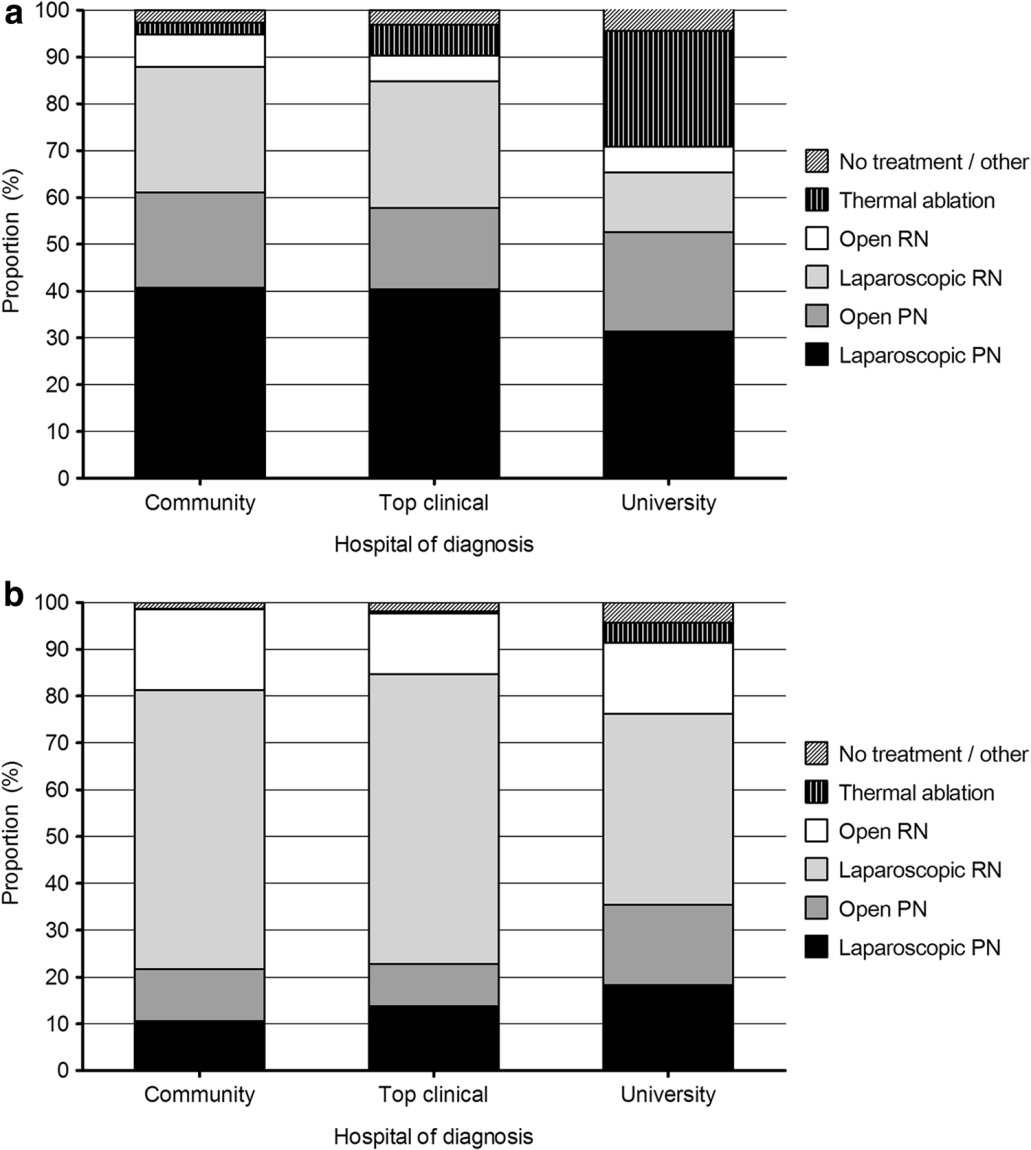
**a** Proportion of cT1a renal cancer patients by treatment modality in the period 2012–2014 by type of hospital of diagnosis. **b** Proportion of cT1b renal cancer patients by treatment modality in the period 2012–2014 by type of hospital of diagnosis

In Fig. 3a, treatment of cT1a renal cancer stratified by hospital type of diagnosis is presented. The majority of patients diagnosed in a university hospital underwent NSS; 53 % PN mostly performed laparoscopically and almost 25 % thermal ablation. Approximately 20 % of all patients had a RN.

Patients diagnosed in the top clinical hospitals and community hospitals were treated slightly different. The majority underwent a PN (57 and 61 %, for top clinical and community hospitals, respectively) and a small proportion of patients had thermal ablation (6.5 and 2.5 %, respectively). A fair part of the patients (>30 %) were still treated with RN.

Patients with a cT1b tumor diagnosed in a university hospital had more often NSS (almost 40 % vs. approximately 20 % for patients diagnosed in top clinical and community hospitals, Fig. 3b). In over 50 % of all patients with a PN, the procedure was done laparoscopically. No large differences were seen between different hospital types. Only 13–17 % of these patients were treated with an open RN.

Multivariable analyses of patients with cT1a (supplementary Table 1) revealed that lower age is associated with a higher probability of PN as well as being treated in a high-volume hospital. In addition, PN was more often performed for tumors in the upper or lower pole of the kidney.

For patients with cT1b, increasing tumor size, tumor localization in the right kidney and being treated in a university hospital were factors significantly associated with a lower probability of a laparoscopic versus open RN in the multivariable analyses. Being treated in a high-volume hospital was associated with a higher probability of laparoscopic surgery (supplementary Table 2).

### Duration of hospital stay and postoperative mortality

Postoperative mortality after cT1a tumor surgery was 0.2 % (*N* = 2, both RN; *p* value *χ*^2^ 0.05) and 0.3 % for cT1b patients (*N* = 4, all RN). Due to small numbers, the association between type and volume of hospital and postoperative mortality could not be studied. Median hospital stay for cT1a patients was 4 days with PN and 4 days with RN for cT1b patients 5 days with PN and 4 days with RN.

## Discussion

A clear trend over time from RN toward PN was observed in patients with cT1a renal cancer; in 2014, the majority was treated according to guideline recommendations with NSS, predominantly PN. The majority of both PN and RN were performed laparoscopically. Limited variation in treatment modalities was observed for patients diagnosed in different hospital types. Most prominent differences were that patients diagnosed with cT1a cancer in a university hospital were more often treated with thermal ablation and less often with RN compared to patients diagnosed in top clinical or community hospitals. Younger age, non-central tumors and being treated in a high-volume hospital were significantly associated with higher probability of PN versus RN for cT1a cancers. Referral from low- to high-volume hospitals for a PN probably partly explains the observed difference between low- and high-volume hospitals.

Concerning cT1b, PN was more frequently performed in recent years, although RN remained the preferred treatment in 2014, with the majority performed laparoscopically. Large difference in proportion of patients treated with NSS was observed for patients diagnosed in a university hospital versus other hospitals; approximately 40 versus 20 % of the patients underwent NSS, respectively. Treated in a high-volume hospital appeared to be strongly associated with a laparoscopical intervention. Again referral to high-volume hospitals specifically for a laparoscopic treatment may be partly responsible.

Our findings largely resemble Swedish data, which is the only recent population-based study on RCC [16]. In Sweden, a significant change over time toward NSS for both cT1a and cT1b renal cell cancers was observed, although the absolute proportion of patients with NSS was lower (62 % of *treated* cT1a patients with NSS vs. 67 % of *all* cT1a patients found in our study). Young age was associated with PN as well. They reported strong variation between university hospitals (62 % NSS for cT1a) and intermediate-volume hospitals with an annual average of 10–30 kidney surgeries (34 %) and low-volume hospitals with <10 kidney surgeries (11 %). This is in line with our findings that university hospitals and high-volume hospitals more often perform NSS.

Studies from the USA based on the National Inpatient Sample evaluating trends over time concerning NSS or PN revealed a clear increase over time, although the general conclusion was that PN remained underused [12, 13, 17]. PN increased from 15 % in 2002 to 25 % in 2008 [13], but in 2008 RN was still applied in 67 % of all patients with cT1 renal cancers [12]. In both studies [12, 13], treatment could not be evaluated by tumor stage, precluding a direct comparison with our study. Another US study based on the SEER database, including only patients with renal tumors ≤4 cm, reported a proportion of 45 % for PN in 2006 [18]. However, though the studies were recently published, the cutoff data for analysis were 7–9 years ago. It is conceivable that in recent years the trend toward PN improved in the USA. Female patients, elderly and those living in rural locations were inversely associated with PN. Similar to our study, in all US studies younger age was significantly associated with PN. Other factors that might be associated with PN included urban hospital location, surgery at teaching hospitals and large hospital capacity [12, 13, 17]. Factors inversely associated with PN were chronic kidney disease and comorbidities (diabetes, hypertension) [12].

As mentioned, it is recommended by the European and US guidelines to treat patients with renal cancers of 4–7 cm (cT1b) with a PN as well, although data from a randomized controlled trial are only available for tumors up to 5 cm. However, the aim to preserve renal function as much as possible has triggered PN in larger tumors despite an increased surgical challenge and tumor size-related risk of progression and survival. From two recent reviews, it can be concluded that survival rates appear to be similar and renal function is better preserved for patients with cT1b renal cancers with PN versus RN [19, 20]. However, current evidence is based on small case series and retrospective observational studies and confounding by indication might have played a role. Despite the advantages of PN in cT1b cancers, the current real-world treatment is (laparoscopic) RN. A likely explanation for this observation is that given the complexity and technical reconstructive skills required for either open, laparoscopic or robot-assisted PN, laparoscopic RN may be easier to learn and offered as the preferred minimally invasive approach.

Our data show that being treated in a university hospital was associated with a lower probability of a laparoscopically RN compared to top clinical and community hospitals. This may be explained by the fact that the overall proportion of cT1b patients treated with RN in university hospitals is significantly lower, because a substantial part is being treated with NSS. Potentially, cT1b cancers not eligible for NSS were the more complex cases resulting in open RN being the preferred treatment.

One limitation of our study was the inclusion of small renal *cancers* only, whereas a part of the small renal masses is *benign*. Therefore, all results only reflect the management of small renal *cancers*. In addition, the slightly better 5-year survival for patients who underwent PN compared to those with RN should not be over-interpreted. Factors such as younger age in patients with PN may have affected the results which demonstrate considerable overlap for the 95 % confidence intervals of the 5-year survival after PN and RN.

 In conclusion, NSS is increasingly applied in the Netherlands. The vast majority of patients are treated according to current guidelines. Compared to a recent analysis from another European country, the Netherlands seems to apply NSS slightly more frequently. Assessment of the adherence to national and European guidelines is of importance to evaluate the quality of health care across member states of the European Union and beyond. Therefore, pre-specified quality indicators and internationally uniform instruments for data collection are needed.

## Electronic supplementary material

Below is the link to the electronic supplementary material.

Supplementary material 1 (DOC 109 kb)

Supplementary material 2 (DOC 115 kb)
