# Supplementary material for: Adherence to guideline recommendations for management of clinical T1 renal cancers in the Netherlands: a population-based study
Source: World J Urol. 2016 May 13;34:1053–60. doi: 10.1007/s00345-016-1841-3 (PMC4958124; doi:10.1007/s00345-016-1841-3)
Supplement: Supplementary file 1 — Supplementary material 1 (DOC 109 kb) [file 345_2016_1841_MOESM1_ESM.doc]

**Tables**

Table 1: Univariable and multivariable logistic regression analyses including patient- and tumor characteristics associated with partial nephrectomy in patients with a cT1a renal cancer treated between 2012-2014.

|  | Partial nephrectomy | Radical nephrectomy | Univariable OR^3^ (95%CI^4^) | Multi-variable OR (95%CI) |
| --- | --- | --- | --- | --- |
| N^1^ | 866 | 461 |  |  |
|  |  |  |  |  |
| Age (mean, sd^4^) | 61.2 (11.0) | 65.5 (10.3) | 0.96 (0.95-0.97) | 0.96 (0.95-0.98) |
|  |  |  |  |  |
| Gender |  |  |  |  |
| Male | 545 (62.9) | 281 (61.0) | Ref |  |
| Female | 321 (37.1) | 180 (39.0) | 0.92 (0.73-1.16) |  |
|  |  |  |  |  |
| Lateralization |  |  |  |  |
| Left | 433 (50.0) | 222 (48.2) | Ref |  |
| Right | 431 (49.8) | 238 (51.6) | 0.93 (0.74-1.16) |  |
| Unknown | 2 (0.2) | 1 (0.2) |  |  |
|  |  |  |  |  |
| Sub-localization |  |  |  |  |
| Central | 172 (19.9) | 134 (29.1) | Ref | Ref |
| Upper/lower pole | 575 (66.4) | 279 (60.5) | 1.61 (1.23-2.10) | 1.61 (1.22-2.11) |
| Not specified | 119 (13.7) | 48 (10.4) | 1.93 (1.29-2.89) | 1.70 (1.12-2.58) |
|  |  |  |  |  |
| Year of treatment |  |  |  |  |
| 2012 | 258 (29.8) | 160 (34.7) | Ref |  |
| 2013 | 290 (33.5) | 149 (32.3) | 1.21 (0.91-1.60) |  |
| 2014 | 318 (36.7) | 152 (33.0) | 1.30 (0.98-1.71) |  |
|  |  |  |  |  |
| Hospital by volume |  |  |  |  |
| < 10 | 48 (5.5) | 44 (9.5) | Ref | Ref |
| 10-19 | 107 (12.4) | 90 (19.5) | 1.10 (0.67-1.79) | 1.17 (0.70-1.94) |
| ≥ 20 | 711 (82.1) | 327 (70.9) | 1.99 (1.30-3.06) | 2.13 (1.29-3.51) |
|  |  |  |  |  |
| Hospital by type |  |  |  |  |
| Community | 251 (29.0) | 163 (35.4) | Ref | Ref |
| Top clinical | 421 (48.6) | 238 (51.6) | 1.15 (0.89-1.48) | 0.80 (0.58-1.10) |
| University | 194 (22.4) | 60 (13.0) | 2.10 (1.48-2.99) | 1.20 (0.79-1.82) |

^1^ N=number

^2^ OR=Odds Ratio

^3^ CI=Confidence Interval

^4^ sd= standard deviation
