# Supplementary material for: Adherence to guideline recommendations for management of clinical T1 renal cancers in the Netherlands: a population-based study
Source: World J Urol. 2016 May 13;34:1053–60. doi: 10.1007/s00345-016-1841-3 (PMC4958124; doi:10.1007/s00345-016-1841-3)
Supplement: Supplementary file 2 — Supplementary material 2 (DOC 115 kb) [file 345_2016_1841_MOESM2_ESM.doc]

Table 2: Univariable and multivariable logistic regression analyses including patient- and tumor characteristics associated with laparoscopic radical nephrectomy (RN) in patients with a cT1b renal cancer treated in 2012-2014.

|  | Laparoscopic RN | Open RN | Univariable OR^2^ (95%CI^3^) | Multi-variable Odds Ratio (95%CI) |
| --- | --- | --- | --- | --- |
| N^1^ | 761 | 191 |  |  |
|  |  |  |  |  |
| Age (mean, sd^4^) | 65.0 (11.1) | 66.9 (11.7) | 0.98 (0.97-1.00) | 0.98 (0.97-1.00) |
|  |  |  |  |  |
| Gender |  |  |  |  |
| Male | 443 (58.2) | 106 (55.5) | 1.0 |  |
| Female | 318 (41.8) | 85 (44.5) | 0.90 (0.65-1.23) |  |
|  |  |  |  |  |
| Tumor size |  |  |  |  |
| 40-49 | 308 (40.5) | 63 (33.0) | 1.0 | 1.0 |
| 50-59 | 238 (31.8) | 61 (31.9) | 0.80 (0.54-1.18) | 0.80 (0.54-1.20) |
| 60-69 | 177 (23.3) | 57 (29.8) | 0.64 (0.42-0.95) | 0.64 (0.42-0.98) |
| Unknown | 38 (5.0) | 10 (5.2) | 0.78 (0.57-1.64) | 1.02 (0.45-2.28) |
|  |  |  |  |  |
| Lateralization |  |  |  |  |
| Left | 388 (51.0) | 78 (40.8) | 1.0 | 1.0 |
| Right | 373 (49.0) | 112 (58.6) | 0.67 (0.49-0.92) | 0.69 (0.49-0.96) |
| Unknown | 0 (0.0) | 1 (0.5) |  |  |
|  |  |  |  |  |
| Sub-localization |  |  |  |  |
| Central/multifocal/overlapping | 174 (22.9) | 46 (24.1) | 1.0 |  |
| Upper/lower pole | 484 (63.6) | 130 (68.1) | 0.98 (0.67-1.44) |  |
| Not specified | 103 (13.5) | 15 (7.9) | 1.82 (0.97-3.41) |  |
|  |  |  |  |  |
| Year of treatment |  |  |  |  |
| 2012 | 262 (34.4) | 72 (37.7) | Ref |  |
| 2013 | 258 (33.9) | 58 (30.4) | 1.22 (0.83-1.80) |  |
| 2014 | 241 (31.7) | 61 (31.9) | 1.09 (0.74-1.59) |  |
|  |  |  |  |  |
| Hospital by volume |  |  |  |  |
| < 10 | 77 (10.1) | 52 (27.2) | 1.0 | 1.0 |
| 10-20 | 174 (22.9) | 27 (14.1%) | 4.35 (2.54-7.44) | 5.06 (2.87-8.92) |
| >20 | 510 (67.0) | 112 (58.6) | 3.08 (2.05-4.62) | 4.30 (2.43-7.61) |
|  |  |  |  |  |
| Hospital by type |  |  |  |  |
| Community | 284 (37.3) | 80 (41.9) | 1.0 | 1.0 |
| Top clinical | 388 (51.0) | 77 (40.3) | 1.42 (1.00-2.01) | 0.80 (0.49-1.30) |
| University | 89 (11.7) | 34 (17.8) | 0.74 (0.46-1.18) | 0.38 (0.20-0.70) |

^1^ N=number

^2^ OR=Odds Ratio

^3^ CI=Confidence Interval

^4^ sd= standard deviation
